# Preventive Effects of Quercetin against the Onset of Atherosclerosis-Related Acute Aortic Syndromes in Mice

**DOI:** 10.3390/ijms21197226

**Published:** 2020-09-30

**Authors:** Masateru Kondo, Yuki Izawa-Ishizawa, Mitsuhiro Goda, Mayuko Hosooka, Yuu Kagimoto, Naoko Saito, Rie Matsuoka, Yoshito Zamami, Masayuki Chuma, Kenta Yagi, Kenshi Takechi, Koichi Tsuneyama, Keisuke Ishizawa

**Affiliations:** 1 Department of Clinical Pharmacology and Therapeutics, Tokushima University Graduate School of Biomedical Sciences, Tokushima 770-8503, Japan; kondou.masateru@tokushima-u.ac.jp (M.K.); uk.exile-namtk511@hotmail.co.jp (Y.K.); r.matsuoka@tokushima-u.ac.jp (R.M.); zamami@tokushima-u.ac.jp (Y.Z.); ishizawa@tokushima-u.ac.jp (K.I.); 2 Department of Pharmacy, Tokushima University Hospital, Tokushima 770-8503, Japan; mgoda@tokushima-u.ac.jp; 3 AWA Support Center, Tokushima University, Tokushima 770-8503, Japan; 4 Department of Medical Pharmacology, Tokushima University Graduate School of Biomedical Sciences, Tokushima 770-8503, Japan; mayu7015@hotmail.co.jp; 5 Department of Pharmacology, Tokushima University Graduate School of Biomedical Sciences, Tokushima 770-8503, Japan; sf3ft5ym7ys9nm@gmail.com; 6 Clinical Research Center for Developmental Therapeutics, Tokushima University Hospital, Tokushima 770-8503, Japan; chuma.masayuki@tokushima-u.ac.jp (M.C.); yagi.kenta@tokushima-u.ac.jp (K.Y.); 7 Department of Clinical Pharmacy, College of Pharmaceutical Sciences, Matsuyama University, Ehime 790-8578, Japan; k.takechi@g.matsuyama-u.ac.jp; 8 Department of Pathology and Laboratory Medicine, Tokushima University Graduate School of Biomedical Sciences, Tokushima 770-8503, Japan; tsuneyama.koichi@tokushima-u.ac.jp

**Keywords:** flavonoids, aortic aneurysm, aortic dissection, endothelial dysfunction, inflammation

## Abstract

Atherosclerosis-related acute aortic syndromes, such as aortic aneurysms or aortic dissection are life-threatening diseases. Since they develop suddenly and progress rapidly, the establishment of preventive strategies is urgently needed. Quercetin, a flavonoid abundant in various vegetables and fruits, is suggested to reduce the risk of cardiovascular disease. Therefore, in this study, the preventive effect of quercetin was evaluated using a mouse model of aortic aneurysm and dissection. The model was established by administering angiotensin II (Ang II) and β-aminopropionitrile (BAPN), a lysyl oxidase inhibitor, to mice to induce hypertension and degeneration of the elastic lamina, which would eventually result in the onset of an aortic aneurysm. Ang II, BAPN, and a nitric oxide synthase inhibitor was administered to induce aortic dissection via endothelial dysfunction. Quercetin (60 mg/kg/day) was administered 2 weeks before inducing aortic diseases by the end of the experiments (8 weeks in the aneurysm model, 6 weeks in the dissection model). It was found to reduce the incidence of aneurysm (from 72 to 45%), dissection (from 17 to 10%), and rupture (from 33 to 15%) in mice. Elastin degradation was ameliorated in the quercetin-treated mice compared to that in the mice without quercetin treatment (degradation score 2.9 ± 0.3 vs 2.2 ± 0.2). Furthermore, quercetin suppressed the expression of vascular cell adhesion molecule-1, macrophage infiltration, and pro-matrix metalloproteinase-9 activity. Our results suggest that quercetin might prevent the onset of atherosclerosis-related acute aortic syndromes through its anti-inflammatory and endothelial cell-protective effects.

## 1. Introduction

Aortic aneurysms and aortic dissection are among the atherosclerosis-related acute aortic syndromes [1,2,3]. An aneurysm is a condition in which a part of the wall of the aorta circumferentially or locally enlarges or protrudes [4]. The known causes of aortic aneurysms are atherosclerosis, hypertension, and weakening of elastic lamina. An ultrasonic screening study revealed the prevalence of abdominal aortic aneurysms among individuals aged 65 years and higher is 4–7% in men and 1–2% in women [5]. In most cases, patients are asymptomatic initially but have a fatal outcome once the aneurysm ruptures [4]. Thus far, only antihypertensive agents have been used therapeutically against aortic aneurysm enlargement or aortic rupture [4].

Aortic dissection is a condition in which the aortic wall is separated into two layers at the level of the media, resulting in the formation of a true lumen and false lumen [4]. Although the cause of dissection is considered to be similar to that of aneurysms, endothelial dysfunction also plays an important role in the onset of dissection [6,7]. In our previous study, we established a novel aortic dissection-prone model mouse that is pharmacologically induced by loading angiotensin II (Ang II) and β-aminopropionitrile (BAPN), a lysyl oxidase inhibitor, to cause hypertension and degeneration of the elastic lamina, respectively, resulting in the onset of an aortic aneurysm [8]. The addition of *N*ω-nitro-l-arginine methyl ester (l-NAME), a nitric oxide synthase (NOS) inhibitor, to this aneurysm model increases the incidence of aortic dissection and aortic rupture due to endothelial dysfunction [6]. We have previously reported that the lipid-lowering drug pitavastatin can inhibit the onset of aortic dissection through its endothelium-protecting and anti-inflammatory effects [6].

Both aortic dissection and aortic rupture due to aneurysms develop extremely rapidly and are associated with a high probability of death [9]. Therefore, we consider that the most important and effective strategy against these atherosclerosis-related acute aortic syndromes is to establish preventive measures that can be effective before the onset of diseases. Several epidemiological studies have shown that a healthy flavonoid-rich diet could help prevent cardiovascular diseases [10,11]. 

Quercetin, a flavonoid abundant in vegetables and fruits, such as onions, shows a potent antioxidative effect [12]. As with other flavonoids, quercetin is thought to be protective against cardiovascular diseases through its endothelium-protecting and anti-inflammatory effects [13,14]. It is considered that most quercetin exists in the plasma as a metabolite that has undergone glucuronidation or sulfate conjugation. Among these metabolites, quercetin-3-O-β-D-glucuronide (Q3GA) has been reported to have antioxidative effects similar to those of quercetin [15]. In our previous study, we reported that Q3GA inhibits vascular smooth muscle cell proliferation and migration via its inhibitory effects on mitogen-activated protein (MAP) kinase activity in vitro [16]. In the present study, we examined the preventive effects of quercetin, for in vivo experiments, or Q3GA, for in vitro experiments, on the onset of aortic aneurysm or dissection.

## 2. Results

The preventive effects of quercetin against aortic aneurysm or dissection were examined using two different mouse models, which were designated as AB, by taking the acronym of two administered drugs; angiotensin II (Ang II) + β-aminopropionitrile (BAPN)-treated aortic aneurysm model mice, and LAB, by taking the acronym of three administered drugs; *N*ω-nitro-l-arginine methyl ester (l-NAME) + Ang II + BAPN-treated aortic dissection model mice.

### 2.1. Quercetin Suppresses Aortic Aneurysm Onset via Anti-Inflammatory Effects

**Figure 1:**
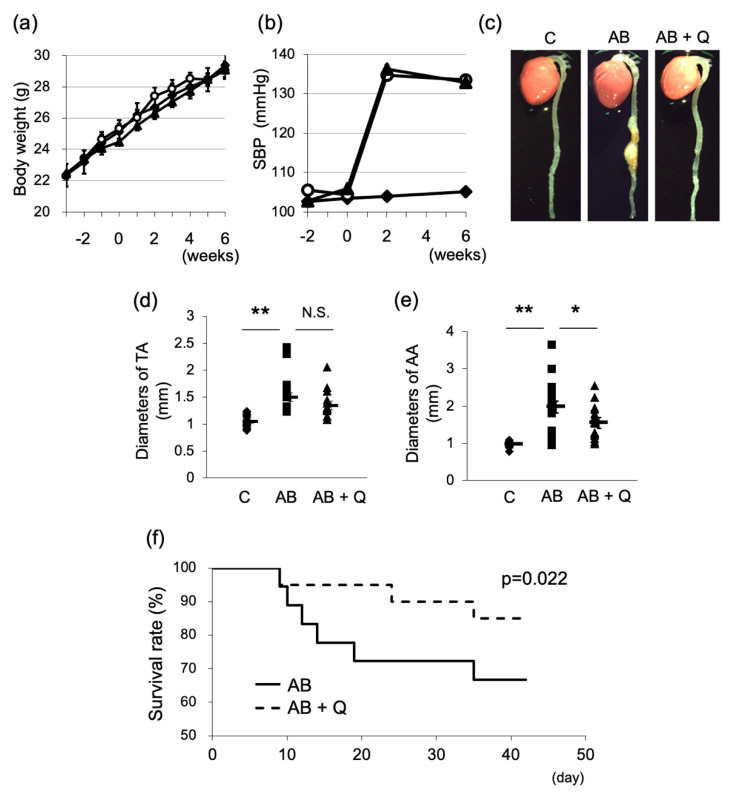
The effects of quercetin administration on aneurysm model mice. C, control; AB, angiotensin II (Ang II) + β-aminopropionitrile (BAPN) treated group; Q, quercetin; SBP, systolic blood pressure; TA, thoracic aorta; AA, abdominal aorta. Week 0 or day 0 indicate the starting day of Ang II plus BAPN loading. The values of body weight (**a**) and SBP (**b**) were mean±S.E. (solid diamond: control, open circle: AB group, solid triangle: AB + Q group). Panel (**c**) shows representative appearance of aortas. The maximum diameters of TA (**d**) and AA (**e**) are shown. Solid bars show means ± S.E. *n* = 18–20. Values are statistically analyzed using two-way analysis of variance (ANOVA) for repeated measures (**a**,**b**,**d**,**e**). * *p* < 0.05, ** *p* < 0.01. (**f**) Survival rates were analyzed by Kaplan–Meier method.

Quercetin treatment was performed for 8 weeks from 2 weeks before the induction of an aneurysm until the end of the experiments. Quercetin treatment did not affect body weight and systolic blood pressure compared to the AB group without quercetin throughout the experimental period (Figure 1a,b). As shown in Figure 1c, more aortas from the quercetin-treated mice showed normal vasculature compared to the aortas from AB mice. Quercetin significantly suppressed the enlargement of the abdominal aortic diameter (Figure 1e) and reduced the incidence of aortic aneurysms (from 72% in the AB group to 45% in the quercetin group) and death from rupture (from 33 to 15%) (Table 1). Survival rate was significantly improved in the quercetin-treated group (Figure 1f). 

**Figure 2:**
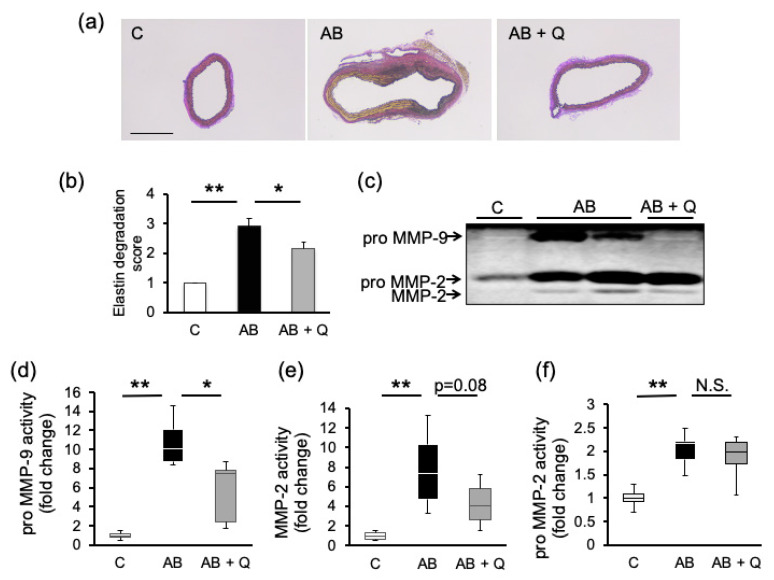
The effects of quercetin on elastin degradation. (**a**) The representative pictures of Elastic- Van Gieson (EVG) staining (bar: 0.5 mm). (**b**) The average values of elastin degradation score estimated under EVG staining (*n* = 13–20). (**c**) The representative figures of zymography assay. Quantified intensity of each band is shown in (**d**), (**e**), and (**f**) (*n* = 4–8). Values are shown as fold increase to the average of control and expressed as mean±SE. Statistical analyses were performed using two-way ANOVA for repeated measures and Bonferroni post hoc test. * *p* < 0.05, ** *p* < 0.01. AB, Ang II + BAPN; Q, quercetin; MMP, matrix metalloproteinase.

The effects of quercetin on the degeneration and collapse of elastic laminae in the aortic aneurysm model mice were assessed based on the degradation score under Elastic-Van Gieson (EVG) staining (Figure 2a). Compared to the control group (average score: 1.0 ± 0.0), the AB group showed enhanced elastin degradation (average score: 2.9 ± 0.3), which was significantly suppressed by quercetin administration (average score: 2.2 ± 0.2) (Figure 2b). The activity of pro-matrix metalloproteinase (MMP)-9, which is abundant in macrophages, was significantly inhibited by quercetin in the aorta compared to that in the AB group (Figure 2c,d). On the other hand, there was no significant difference in the activity of MMP-2 and pro-MMP-2, which is abundant in vascular smooth muscle cells (Figure 2e,f).

**Figure 3:**
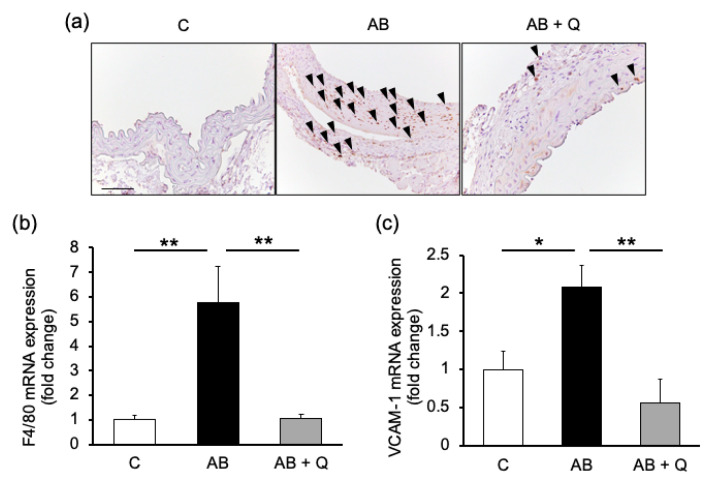
The effects of quercetin on inflammatory responses in aortas. Panel (**a**) shows representative pictures of Mac-2 expressions. Arrow heads are positive staining. Bar: 0.1 mm. Graphs show the mRNA expressions of inflammatory markers, F4/80 (**b**) and vascular cell adhesion molecule (VCAM)-1 (**c**) in aortas. *n* = 4–6. Values are shown as fold increase to the average of control and are expressed as mean±SE. Statistical analyses were performed using two-way ANOVA for repeated measures and Bonferroni post hoc test. * *p* <0.05, ** *p* <0.01. AB, Ang II + BAPN; Q, quercetin.

Furthermore, quercetin suppressed macrophage infiltration into the aortic wall, as indicated by the immunostaining of Mac-2 (Figure 3a) and mRNA expression of F4/80 (Figure 3b). These findings were consistent with the results that pro-MMP-9 was suppressed in quercetin treated aortas, as shown in Figure 2d. The expression of vascular cell adhesion molecule (VCAM)-1, which can recruit inflammatory cells including macrophages, was also suppressed by quercetin treatment (Figure 3c).

### 2.2. Quercetin Shows Endothelial Cell-Protective Effects in Cultured Human Umbilical Vein Endothelial Cells (HUVECs)

**Figure 4:**
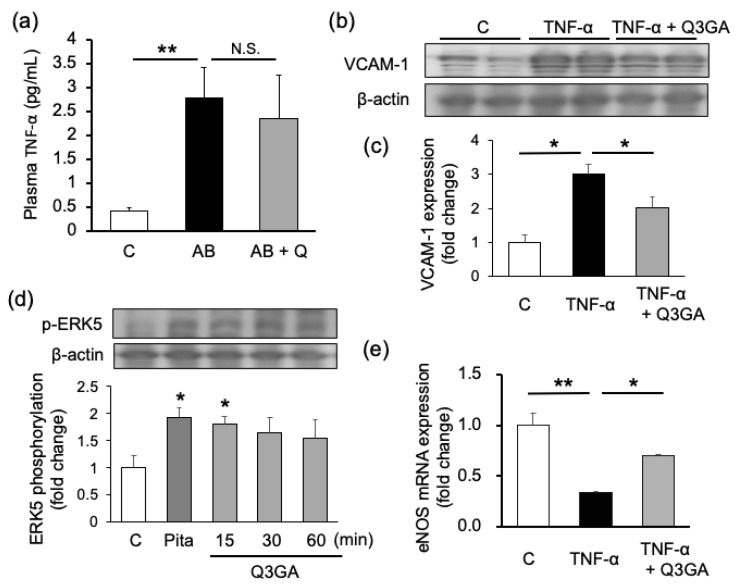
The effects of quercetin on plasma tumor necrosis factor (TNF)-α concentration and the quercetin-3-O-β-D-glucuronide (Q3GA) effects in cultured human umbilical vein endothelial cells (HUVECs). (**a**) TNF-α concentration in plasma from AB group mice (*n* = 5–8). Protein expression of VCAM-1 and extracellular signal-regulated kinase (ERK) 5 phosphorylation in HUVECs were analyzed by Western blotting. Representative bands ((**b**) and upper panel of (**d**)), and quantified intensity ((**c**) and lower panel of (**d**)) are shown. The mRNA expression of endothelial nitric oxide synthase (eNOS) in HUVECs are shown in (**e**). *n* = 4–6. Values are shown as fold increase to the average of control and are expressed as mean±SE. Statistical analyses were performed using two-way ANOVA for repeated measures and Bonferroni post hoc test. * *p* < 0.05, ** *p* < 0.01 (vs. control in (**d**)). AB, Ang II + BAPN; Q, quercetin; Pita, pitavastatin.

Tumor necrosis factor (TNF)-α, an inflammatory cytokine, potently induces VCAM-1 [17]. TNF-α in mouse plasma was significantly increased in the AB group (average: 2.79 pg/mL) as compared to the control group (average: 0.42 pg/mL). There was no difference between the AB group and quercetin-treated group (average: 2.35 pg/mL) (Figure 4a). Therefore, we examined the effect of quercetin on TNF-α-induced signaling in cultured human umbilical vein endothelial cells (HUVECs), which is well established for research on TNF-α signaling to induce VCAM-1 expression [17,18], using quercetin-3-O-β-D-glucuronide (Q3GA), a quercetin metabolite. VCAM-1 expression was increased by TNF-α stimulation and suppressed both by quercetin and Q3GA (Figure 4b). Endothelial NOS (eNOS), which is an important molecule for maintaining endothelial cell function, was downregulated by TNF-α stimulation, but recovered by Q3GA pretreatment (Figure 4d). Moreover, Q3GA, as well as pitavastatin, a positive control, phosphorylated extracellular signal-regulated kinase (ERK) 5, which is known as an endothelial cell-protective molecule and induces eNOS expression [19] in HUVECs (Figure 4c). 

### 2.3. Quercetin Suppresses the Incidence of Aortic Dissection and Mortality in Mouse Models of Dissection

**Figure 5:**
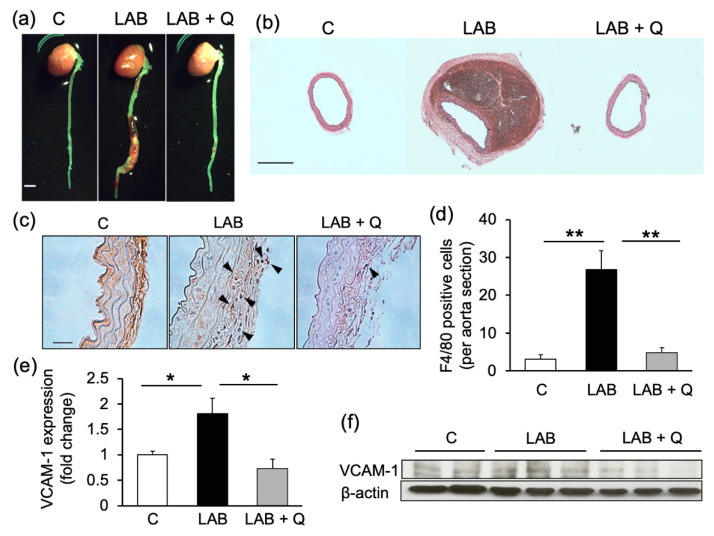
The effects of quercetin on aortic dissection model. The representative pictures of gross aortas (**a**, bar: 1.0 mm) and EVG staining (**b**, bar: 0.5 mm). The representative pictures of immunostaining by F4/80 (**c**, bar: 100 µm), and the quantified graph (**d**) are shown. Protein expression of VCAM-1 in mice aortas were analyzed by Western blotting. Representative bands (**f)** and quantified intensity (**e**) are shown. *n* = 4–6. Values are shown as fold increase to the average of control and are expressed as mean±SE. Statistical analyses were performed using two-way ANOVA for repeated measures and Bonferroni post hoc test. * *p* < 0.05, ** *p* < 0.01. LAB, l-NAME + Ang II + BAPN; Q, quercetin.

Since the effect of quercetin on endothelial dysfunction was observed (Figure 4), it was hypothesized that quercetin is more effective against aortic dissection, which is highly associated with endothelial dysfunction. The effect of quercetin in the aortic dissection model (LAB group) was examined. The effect of quercetin on the incidence of aortic dissection or rupture was assessed 1 week after Ang II + BAPN loading. Figure 5a,b show representative pictures of macroscopic figures and EVG staining of mouse aortas, respectively. The incidence of aortic dissection reduced to 16% in the quercetin-treated group, compared to 33% in the LAB group. Twenty-two percent of the mice in the LAB group died from aortic rupture, whereas none died in the quercetin-treated group (Table 2).

The effect of quercetin on the inflammatory response in the aortic dissection model was examined. Macrophage infiltration into the aortic wall, which was assessed by immunostaining of F4/80, increased in the LAB group and was suppressed by quercetin treatment (Figure 5c,d). VCAM-1 expression in the aorta was also upregulated in the LAB group and was suppressed by quercetin treatment (Figure 5e,f).

## 3. Discussion

In the present study, we suggested that quercetin administration might prevent atherosclerosis-related acute aortic syndromes, such as aortic aneurysm, dissection, and death from rupture, in mice. The preventive effect of quercetin against these conditions was attributable to its anti-inflammatory and endothelial cell-protective effects, which were independent of its blood pressure-lowering effect. 

The pathophysiological features of aortic aneurysms and dissection are very similar and are due to atherosclerosis, hypertension, and weakened elastic lamina. Since the collapse of the endothelium is essential for the onset of dissection or aortic rupture, the involvement of endothelial dysfunction is more strongly suspected in aortic dissection or rupture compared to that in aneurysms. As shown in Table 1, quercetin suppressed the incidence of aortic aneurysms from 72 to 45%; however, nine out of 20 mice in the aneurysm-prone AB group still showed aneurysm formation. On the other hand, quercetin decreased the incidence of dissection and rupture from six and four out of 18 to four and zero out of 25 mice, respectively, in the dissection-prone LAB group (Table 2). In the present study, the number of animals used in the experiment was minimal; therefore, a significant difference was detected only in terms of a suppressive effect on aortic rupture. However, there was a clear tendency of prevention of the onset of aortic dissection. These results suggest that quercetin showed a stronger effect in the LAB group than in the AB group (Table 1 and Table 2) through its endothelial protective effect. 

In a previous study, we reported that an increase in nitric oxide may upregulate the expression of the cell junction molecule, vascular endothelial cadherin, via ERK5 expression and protect the endothelium from vascular hyperpermeability [6]. Pitavastatin phosphorylates ERK5 directly and activates its downstream pathways including eNOS expression [6]. In the present study, we have showed that quercetin activated ERK5 to the same extent as pitavastatin (Figure 4). Moreover, several studies have demonstrated that flavonoids such as fisetin and puerarin also activate the ERK5/MEF2c/KLF2 pathway—which induces eNOS expression—and show endothelial cell protective effects [20,21]. From these reports, it is strongly suggested that quercetin’s effect is mediated by the ERK5 pathway. Further examination is needed to clarify the detailed molecular pathway.

Moreover, we found that quercetin could suppress VCAM-1 expression in mouse aorta and cultured HUVECs (Figure 3, Figure 4 and Figure 5). This finding was consistent with that reported by Lee et al., showing that quercetin suppressed vascular endothelial growth factor-induced inflammatory responses, such as the translocation of nuclear factor kappa B, the upregulation of adhesion molecules and MMPs, and the downregulation of tight junction molecules [22]. In the present series of experiments, the tendency was shown that VCAM-1 expression in aorta tissue was lower in the quercetin treated sample than control sample. This tendency was observed in both mRNA expressions in aneurysm mice (Figure 3c) and protein expressions in dissection mice (Figure 5e,f), but not the expression in HUVECs (Figure 4b,c). From these results, it was implied that sham operation or the daily administration of vehicle might have affected to basal systemic inflammatory response. Actually, other reports also showed relatively high expression of VCAM-1 in control with sham operation [23]. In the present study, quercetin was administered with high concentration, so that the expression of VCAM-1 could be suppressed to the lower level compared to the sham-treated control. Infiltrated macrophages via association with VCAM-1 play an important role in the degradation of elastic lamina through the activation of MMP-9, an extracellular matrix-degrading enzyme [24]. Since quercetin treatment significantly inhibited macrophage infiltration into the aortic wall (Figure 3), pro-MMP-9 activity also significantly decreased in the quercetin-treated mice (Figure 2). 

In the present study, we demonstrated the suppressive effects on elastin degradation via the inhibition of macrophage infiltration but not via direct action to aortic media. However, we have found that quercetin also affects vascular smooth muscle cell function in the progression of cardiovascular disease in the previous study. The quercetin derivative Q3GA inhibited vascular smooth muscle cell proliferation and migration via its inhibitory effects on MAP kinase activity [16]. Pereira et al. demonstrated that quercetin decreases the activity of MMP-2 and ameliorates vascular remodeling in rats with renovascular hypertension [25]. Therefore, it is speculated that quercetin acts on both the intima and media of the aorta to suppress the onset of aortic aneurysm and dissection. Future studies need to clarify the effect of quercetin on vascular smooth muscle cells in the progression of atherosclerosis-related acute aortic syndrome.

A recent systematic review and meta-analysis on the effect of quercetin against cardiovascular diseases demonstrated that long-term supplementation with quercetin could lower blood pressure, high-density lipoprotein cholesterol, and triglycerides in humans [26]. In addition, it is very meaningful that our study showed that quercetin supplementation could prevent even sudden cardiovascular events. In this study, however, high doses of quercetin (60 mg/kg/day, via p.o.) that cannot be taken with a normal diet are administered. Therefore, the continuous intake of quercetin as a medication could be beneficial, especially for those at high risk of cardiovascular events. Further clinical investigations of the effects of quercetin on atherosclerosis-related acute aortic syndrome and application to new drug developments are desired.

## 4. Materials and Methods

### 4.1. Ethics Statement

This study conformed to the Guide for the Care and Use of Laboratory Animals (NIH Publication No. 85–23, 1996) [27]. All animal procedures were performed in accordance with the guidelines of the Animal Research Committee of the University of Tokushima Graduate School, and the protocols were approved by the Tokushima University Institutional Review Board for animal protection; approval No. is T30-85.

### 4.2. Reagents

Angiotensin II (Ang II), β-aminopropionitrile (BAPN), *N*ω-nitro-l-arginine methyl ester (l-NAME), quercetin, quercetin-3-O-β-D-glucuronide (Q3GA), and tumor necrosis factor-α (TNF-α) were purchased from Sigma-Aldrich Japan (Tokyo, Japan). β-actin (3700s) antibodies were purchased from Cell Signaling Technology Inc. (MA, USA). VCAM-1 (E-10) antibody and F4/80 (M-300) antibody were purchased from Santa Cruz Biotechnology Inc. (CA, USA). Mac2 antibody was from Cedarlane Laboratories Ltd. (NC, USA). All other reagents and instruments we used in the present study are commercially available.

### 4.3. Mice and In Vivo Experimental Strategies

C57BL/6J male mice (6-8 weeks old, weighing 20–25 g) were purchased from CLEA Japan Inc. (Tokyo, Japan). Sixty mice were used for each of the aortic aneurysm experiments and the dissection experiment in this study in accordance with statistically estimated sample sizes and divided to three groups in a random manner. The animals were housed in a temperature-controlled room at 25 °C under a 12-h light/dark cycle with free access to food and water. C57BL/6 J mice were anesthetized by an intraperitoneal injection of 100–150 mg/kg sodium pentobarbital, and more was administered if the mice moved in response to pain after 30 min from the start of the operation. Pedal withdrawal reflex, toe pinch reflex, muscular relaxation, and respiratory rates were monitored to ensure that adequate anesthesia was administered. Aneurysm model mice and dissection model mice were created as previously reported [16,28]. Briefly, in the aortic aneurysm model, 11-week-old mice were implanted dorsally with two subcutaneous osmotic mini-pumps (Model 2006 2002 Micro-osmotic Pump; Alzet, Cupertino, CA, USA) to administer Ang II (1000 ng/kg/day for 6 weeks) and BAPN (150 mg/kg/day for 2 weeks). For development of dissection mice, l-NAME (10 mg/kg/day) was orally administered in drinking water from 8 weeks of age until the end of the experiment. After 3 weeks from the start of experiment, Ang II (1000 ng/kg/day) and BAPN (150 mg/kg/day) were administered for 1 week. Each group was abbreviated as below:

C—control, untreated group;

AB—Ang II + BAPN treated aneurysm group;

LAB—l-NAME + Ang II + BAPN treated dissection group.

Quercetin was administered daily via a feeding needle at the dose of 60 mg/kg/day based on the previous study [29] from 2 weeks before the start of other drug administration to the end of experiment; continued for 8 weeks in the aneurysm model or 6 weeks in the dissection model.

### 4.4. Systolic Blood Pressure (SBP)

SBP was measured in conscious mice by tail-cuff plethysmography (BP-98A, Softron, Tokyo, Japan).

### 4.5. Tissue Sampling and Measurement of Aortic Diameter

At the endpoint of the experiment, the animals were anesthetized by intraperitoneal injection of sodium pentobarbital (150 mg/kg or more) and euthanized by cervical dislocation. The whole heart and aorta were isolated and photographed by SZ61 Olympus stereomicroscope (Olympus Corp., Tokyo, Japan). Maximum diameters of aortas were measured using ImageJ v. 1.37 software (National Institutes of Health, Bethesda, MD, USA). Anomalies where the abnormal site had expanded beyond 1.5-times the normal diameter were judged as aneurysms, and the aortic aneurysm incidence rate was determined. Blood samples were incubated at 37 °C for 30 min and centrifuged at 2600× *g* for 2 min. The supernatant serum was used to measure the TNF-α. For the morphometry, the aortas were resected and placed in 10% buffered formalin. After fixation, the tissues were embedded in paraffin.

### 4.6. Histology and Immunohistochemistry

Sections (5 μm thickness) were subjected to Elastic-van Gieson (EVG) staining (Elastic-van Gieson staining kit; MERCK, Kenilworth, NJ, USA) according to the manufacturer’s instructions. The onset of aortic dissection was determined by the formation of false lumen under EVG staining. Elastin degradation was graded as follows: grade 1, intact, well-organized elastic laminae; grade 2, elastic laminae with some interruptions and breaks; grade 3, severe elastin fragmentation or loss [28]. Immunohistochemistry was performed using Mac-2 antibody (1:200 dilution) and F4/80 antibody (1:50dilution), as previously described. Peroxidase (for Mac-2) and Mayer hematoxylin (for F4/80) were used for counterstaining. Measurements were taken using Image J v. 1.37 software.

### 4.7. Quantitative Real-Time PCR

The mRNA expression levels in the aortas or culture cells were analyzed by real-time PCR, as described previously [6]. Sequences of the amplification primer pairs are as shown in Table 3. 

### 4.8. Zymography Assay

MMP activities in aortic tissues were measured by zymography assay, as previously described [30]. Briefly, frozen aortas were homogenized by polytron in buffer containing 10 mM CaCl2 and 0.25% Triton-X100. Homogenates in sample buffer (250 mM Tris-HCl (pH 6.8) with 100 mg/mL SDS, 35% grycerol, and 0.05% bromophenol blue) were subjected to electrophoresis in a 10% acrylamide gel containing 1 mg/mL gelatin. After shaking in a regeneration buffer (2.5% Triton-X100 solution) for 1 h, enzymatic reaction was performed in a buffer (50 mM Tris-HCl (pH 7.6) with 10 mM CaCl2 and 1% Triton-X100) at 37 °C for 20 h. Thereafter, protein staining (Coomassie Brilliant Blue R) was performed. The intensities of bands were analyzed using ImageJ v. 1.37 software.

### 4.9. ELISA for TNF-α Measurement

TNF-α in mouse plasma was quantified using the Quantikine Mouse TNF-α ELISA Kit (R & D systems, Minneapolis, MN, USA) according to the manufacturer’s instructions. Briefly, sample plasma or standard peptide were reacted with Diluent Buffer at room temperature for 2 h and followed by the reaction with conjugate for 2 h and substrate solution for 30 min sequentially. After stopping the reaction, the absorbance was measured at 450 nm.

### 4.10. Cell Culture

Human umbilical vein endothelial cells (HUVECs) were purchased from Lonza Japan Inc., which confirmed the cell quality by the expressions of von Willebrand factor, Factor VIII, CD31 and CD105. All cells were cultured in Endothelial Cell Growth Medium (EGM)-2 Bullet Kit (Lonza Japan Inc, Tokyo, Japan) with 4% fetal bovine serum. Passage 4–6 cells were grown to confluence and treated with the quercetin derivatives Q3GA or TNF-α for indicated time and concentrations. Cell lysates were subjected to Western blotting or real time PCR analysis.

### 4.11. Western Blotting

Western blotting analysis for protein expression and ERK5 phosphorylation in mice aortas or HUVECs was performed as described previously [6]. Briefly, lysates of aortas or HUVECs were subjected to sodium dodecyl sulphate-polyacrylamide gel electrophoresis and electro-transferred to polyvinylidene difluoride membranes, as described previously. The membranes were blocked in phosphate-buffered saline with 0.1% Tween-20 (PBS-T), containing 4% milk powder for 90 min at room temperature and then incubated overnight at 4 °C with one of the following primary antibodies: VCAM-1 antibody (sc-8304; Santa Cruz, Santa Cruz, CA, USA), phospho-ERK5 antibody (#3371; Cell Signaling, Danvers, MA, USA), ERK5 antibody (#3372; Cell Signaling), or β-actin antibody (as a loading control, #3700; Cell Signaling). The membranes were subsequently incubated with horseradish peroxidase-conjugated anti-rabbit IgG (GE Healthcare Biosciences, Piscataway, NJ, USA) for 1 h at room temperature, and the signals were developed using ECL Plus Western Blotting Detection System (GE Healthcare Biosciences, Piscataway, NJ, USA). Immunoreactive bands were quantified by densitometry in the linear range of film exposure using a UMAX Astra 2200 scanner and image J v. 1.37 software (National Institutes of Health, Bethesda, MD, USA). 

### 4.12. Statistical Analysis

The results of the experiments were presented as means ± standard error (SE). Statistical analysis was performed using StatMate IV for Windows (Atoms, JAPAN). The normally distributed continuous variables for more than 3 groups were compared using two-way ANOVA and for 2 groups were compared using *t*-test. Non-normal distribution continuous variables were compared using Kruskal–Wallis test and further paired comparisons were done using Mann–Whitney U test. Nominal categorical data between the groups were compared using Chi-squared (χ2) or Fisher’s exact test as appropriate. For all statistical tests, a *P* value less than 0.05 and 0.01 was taken to indicate a significant and highly significant difference, respectively. 

## Figures and Tables

**Table 1:** Incidence of aortic aneurysms and rupture.

	Control, *n* (%)	AB, *n* (%)	AB + Q, *n* (%)	*p* Value *
**TAA**	0/18 (0%)	3/18 (17%)	2/20 (10%)	0.653
**AAA**	0/18 (0%)	13/18 (72%)	9/20 (45%)	0.090
**total aneurysm**	0/18 (0%)	13/18 (72%)	9/20 (45%)	0.090
**rupture**	0/18 (0%)	6/18 (33%)	3/20 (15%)	0.26

* AB vs. AB + Q by Fisher’s exact test or Pearson’s chi-square test. Abbreviations are as below: AB, Ang II + BAPN; Q, quercetin; TAA, thoracic aortic aneurysm; AAA, abdominal aortic aneurysm.

**Table 2:** Incidence of aortic dissection and rupture.

	Control, *n* (%)	LAB, *n* (%)	LAB + Q, *n* (%)	*p* Value
**Dissection**	0/17 (0%)	6/18 (17%)	4/25 (10%)	0.275
**rupture**	0/17 (0%)	4/18 (33%)	0/25 (15%)	0.025 *

* LAB vs. LAB + Q by Fisher’s exact test. Abbreviations are as below: LAB, l-NAME + Ang II + BAPN; Q, quercetin.

**Table 3:** Sequence of the primers used for real-time PCR.

Target gene	Forward (5’-3’)	Reverse (5’-3’)
*mF4/80*	CTTGGCTATGGGCTTCCAGTC	GCAAGGAGGACAGAGTTTATCGTG
*mVCAM1*	CCATTGAAGATACCGGGAAAT	TAGCTGTCTGCTCCACAGGAT
*mβactin*	AAGTGTGACGTTGACATCCG	GATCCACATCTGCTGGAAG
*hVCAM1*	GGTGGGACACAAATAAGGGTTTTGG	CTTGCAATTCTTTTACAGCCTGCC
*heNOS*	TGGTACATGAGCACTGAGATCG	CCACGTTGATTTCCACTGCTG
*hβactin*	GCGGGAAATCGTGCGTGACATTA	ATGGAGTTGAAGGTAGTTTCGTG
